# Hip decompression of unstable slipped capital femoral epiphysis: a systematic review and meta-analysis

**DOI:** 10.1007/s11832-015-0648-x

**Published:** 2015-03-17

**Authors:** Talal Ibrahim, Shady Mahmoud, Muhammad Riaz, Abdelsalam Hegazy, David G. Little

**Affiliations:** 1 Department of Orthopedic Surgery, Hamad General Hospital, Weill Cornell Medical College in Qatar, P.O. Box 3050, Doha, Qatar; 2 Department of Orthopaedic Surgery, Hamad General Hospital, Doha, Qatar; 3 Population Health Research Institute, St George’s, University of London, London, UK; 4 Orthopaedic Research and Biotechnology Unit, The Children’s Hospital at Westmead, Westmead, NSW 2145 Australia

**Keywords:** Slipped capital femoral epiphysis, Unstable, Osteonecrosis, Decompression, Meta-analysis

## Abstract

**Purpose:**

Slipped capital femoral epiphysis (SCFE) is one of the most common adolescent hip conditions. Unstable SCFE is characterized by sudden and severe hip pain with the inability to weight bear, even with crutches. Osteonecrosis of the femoral head is increased in patients with unstable SCFE. The aim of our study was to systematically review the literature that compares hip decompression to no hip decompression of unstable SCFE.

**Methods:**

We searched several databases from 1946 to 2014 for any observational or experimental studies that evaluated hip decompression and osteonecrosis of unstable SCFE. We performed a meta-analysis using a random effects model to pool odds ratios (ORs) for the comparison of osteonecrosis between patients undergoing hip decompression and no hip decompression. We also investigated the type of hip decompression performed. Descriptive, quantitative, and qualitative data were extracted.

**Results:**

Of the 17 articles identified, nine studies (eight case series and one retrospective cohort study) were eligible for the meta-analysis, with a total of 302 unstable SCFE. The pooled OR = 0.91 of osteonecrosis between hip decompression and no hip decompression was in favor of hip decompression, but was not statistically significant [95 % confidence interval (CI): 0.47, 1.75; *p* = 0.54, *I* ^2^ = 0 %]. No significant differences in the rates of osteonecrosis were detected in unstable SCFE with open and percutaneous hip decompression alone (OR = 0.97, 95 % CI: 0.36, 2.62; *p* = 0.69, *I* ^2^ = 19.1 %) or hip decompression with bony procedures (OR = 0.99, 95 % CI: 0.35, 2.79; *p* = 0.69, *I* ^2^ = 0 %).

**Conclusions:**

The cumulative evidence at present does not indicate an association between hip decompression and a lower rate of osteonecrosis of unstable SCFE. However, hip decompression of unstable SCFE remains an option that can potentially decompress the intracapsular hip pressure and optimize the blood flow to the femoral head. Thus, multicenter prospective cohort studies are required and will be able to answer this question with more certainty and a higher level of evidence.

**Level of evidence:**

Level III/IV.

## Introduction

Slipped capital femoral epiphysis (SCFE) is one of the most common adolescent hip conditions that have potential long-term sequelae and are dependent on the severity of the SCFE [1]. SCFE can be classified into two groups: stable and unstable according to Loder et al. [2]. A stable SCFE is defined as one where the patient is able to ambulate, with or without crutches, regardless of the duration of symptoms, whereas an unstable SCFE is defined as one where the patient cannot ambulate, with or without crutches, regardless of the duration of symptoms. This classification system is the most useful because of its correlation with prognosis by estimating the risk of osteonecrosis of the femoral head that was reported as 47 % for unstable SCFE in this classic paper [2].

The management of unstable SCFE remains controversial, with little consensus on the best treatment and with most recommendations made with level IV evidence [3]. However, in situ pinning remains the gold standard for the management of unstable SCFE [4]. The literature suggests that urgent reduction with internal fixation and decompressive arthrotomy results in the lowest rate of osteonecrosis of the femoral head for unstable SCFE [3]. The current controversies regarding the treatment of unstable SCFE include the role of hip decompression, the timing of intervention, the role of reduction, the type of surgical fixation, and the post-operative management. The goals of treatment of unstable SCFE include the avoidance of osteonecrosis and chondrolysis of the femoral head, prevention of further slippage, and correction of proximal femoral deformity. More recently, the role of surgical dislocation in the management of moderate and severe unstable SCFE has become popularized because of the advantage of anatomically correcting the acute proximal femoral deformity and, thus, preventing osteoarthritis secondary to femoroacetabular impingement, with reported rates of osteonecrosis ranging from 0 to 6.7 % [5, 6].

Osteonecrosis of the femoral head is the most significant complication in patients with unstable SCFE. The rate of osteonecrosis varies between studies and has been reported to range from 3 to 58 % [7]. A recent review of unstable SCFE reported an overall rate of osteonecrosis of 23.9 % with multiple treatment modalities used and limited data concerning complications after treatment of unstable SCFE [8]. The etiology of osteonecrosis of the femoral head remains unknown and is most likely multifactorial due to disruption or kinking of the retinacular vessels to the epiphysis or vascular tamponade due to increased intracapsular hip pressure. Kinking of the retinacular vessels has been demonstrated in an angiographic study of unstable SCFE with restoration of blood supply after reduction [9]. Increased intracapsular hip pressure has also been studied in unstable SCFE. Herrera-Soto et al. [10] measured the intracapsular joint pressure in 13 unstable SCFE. The mean intracapsular joint pressure of the unstable SCFE measured 48 mm Hg, which increased to 75 mm Hg after manipulative reduction and dropped to 17 mm Hg after capsulotomy and decompression.

The aim of our study was to systematically review the literature that compares hip decompression to no hip decompression of unstable SCFE. The primary outcome analysis involved the rate of osteonecrosis of the femoral head.

## Materials and methods

### Search strategy

A senior medical librarian with 40 years of experience developed the search strategy and performed the literature search. The databases that were searched included Ovid MEDLINE (1946 to August 2014), Ovid EMBASE (1988–2014), Web of Science, Elsevier Scopus, and the Cochrane Registry of Clinical Trials. The primary terms were “slipped capital femoral epiphysis” combined with “unstable”, “avascular necrosis”, and “osteonecrosis”. Articles were not limited to any particular study design. Two authors independently assessed the eligibility of the identified studies. Any study that could be relevant based on the respective abstract was reviewed in full text. Bibliographies and review articles were reviewed manually for additional citations. The language of the publications was restricted to English. We did not seek unpublished investigations.

### Study selection

We considered any study design that compared hip decompression and no hip decompression of unstable SCFE and reported the rate of osteonecrosis of the femoral head. An unstable SCFE was defined as a patient with pain so severe that walking was not possible even with crutches, regardless of the duration of symptoms according to the Loder et al. [2] classification. Hip decompression was classified into percutaneous and/or open hip capsulotomy or no hip capsulotomy.

### Data collection

Two authors independently extracted and recorded the required datasets, which included study characteristics (i.e., country, year of study), mean age of patients, number of unstable SCFE, method of reduction and treatment of unstable SCFE, and the number of cases of osteonecrosis of the femoral head. Two authors independently assessed the methodological quality of retrospective cohort studies according to key validity components that address selection, comparability, and exposure using the Newcastle–Ottawa Scale [11] to assess the quality of non-randomized studies. Any disagreement was resolved by consensus.

### Statistical methods

We pooled studies and constructed Forest plots using the DerSimonian–Laird random effects model [12], which recognizes studies as a sample of all potential studies and incorporates a between-study random effect component to allow for between-study heterogeneity. Between-study heterogeneity was quantified using the *I* ^2^ statistic. This defines the variability percentage in effect estimates that is due to heterogeneity rather than to chance; the larger the *I* ^2^, the greater the heterogeneity.

We based the main meta-analytic comparison on the odds ratio (OR) of the osteonecrosis rate in patients undergoing hip decompression versus those undergoing no hip decompression. The osteonecrosis rate was obtained by dividing the number of unstable SCFE that developed osteonecrosis of the femoral head by the total number unstable SCFE. The diagnosis of osteonecrosis was defined by clinical and radiological findings. If no event occurred in at least one cell of the (2 × 2) contingency table for a parent study, a continuity correction of 0.5 was added to each cell to compute the OR and permit analysis, as described in the Cochrane handbook [13].

A further sensitivity analysis was performed with the inclusion of the study by Kallio et al. [14]. Additional sensitivity analyses were performed to determine the rate of osteonecrosis in patients who underwent hip decompression with no bony procedures, such as osteotomies, and those who underwent hip decompression with bony procedures.

## Results

### Yield of the search strategy and eligible studies

**Fig. 1:**
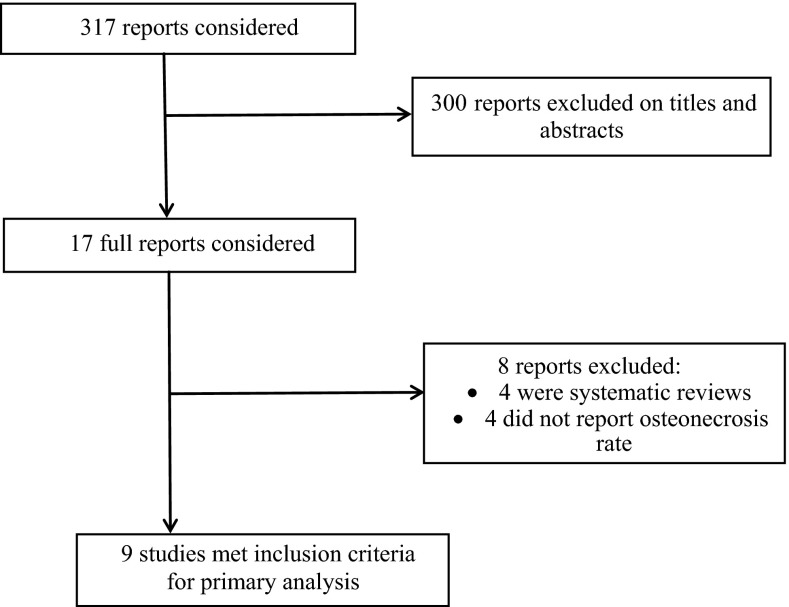
Flow diagram of eligible studies

The search strategy yielded 317 publications, of which we considered 17 articles for full-text review. We excluded four systematic reviews and a further four not fulfilling our inclusion criteria of unstable SCFE and not reporting the rate of osteonecrosis in patients undergoing hip decompression compared to those with no hip decompression. A total of nine studies addressing hip decompression versus no hip decompression were eligible [2, 15–22]. Figure 1 summarizes the process of identifying eligible studies. Eight studies were case series and one was a retrospective cohort study. There were no prospective cohort studies. The kappa statistics for interobserver agreement on study eligibility was 1.0.

### Characteristics of the included studies

Table 1 summarizes the characteristics of the nine studies included in our primary and sensitivity analyses. The studies included a total of 302 unstable SCFE, with a total of 59 cases of osteonecrosis. The individual sample sizes of the studies ranged from 12 to 91 unstable SCFE. The definition of osteonecrosis was similar in all nine studies, utilizing radiographs for evidence of sclerosis and/or collapse. Some of the studies also utilized bone scans to demonstrate a lack of blood supply to the femoral head [15, 17, 18]. The follow-up period of the studies varied, but the shortest follow-up period was 24 months from the date of treatment to record the appearance of osteonecrosis [23]. The overall rate of osteonecrosis for all the included studies was 19.5 %. The majority of cases of osteonecrosis (63 %) occurred in patients that did not undergo hip decompression. In the study by Phillips et al. [20], no cases of osteonecrosis were reported.

**Table 1:** Characteristics of the included studies in the meta-analysis

Source, year, country	Mean age (years)	Unstable SCFE (number)	Treatment/reduction	Number of osteonecrosis cases (decompression:no decompression)	Follow-up (months)
Alves et al. 2012, Canada [15]	12.2	12	6 CR and 6 OR	6 (4:2)	41
Sankar et al. 2010, USA [16]	12.6	70	16 in situ pinning, 38 CR, and 16 OR	14 (5:9)	38
Chen et al. 2009, USA [17]	11.6	30	25 CR and 5 OR	4 (3:1)	65
Gordon et al. 2002, USA [18]	11.1	16	12 CR and 4 OR	2 (1:1)	27
Kennedy et al. 2001, USA [19]	11.3	27	11 in situ pinning, 3 traction, 11 CR, and 2 OR	4 (2:2)	Minimum 24
Phillips et al. 2001, UK [20]	13	14	Gentle manipulation, 2 Dunn osteotomies	0 (0:0)	Minimum 36
Peterson et al. 1997, USA [21]	–	91	4 cast, 41 pinning, 31 epiphysiodesis, and 15 epiphysiodesis and pins	13 (16:7)	Minimum 84
Aronson and Tursky 1996, USA [22]	–	12	3 CR and 9 OR	2 (0:2)	Minimum 24
Loder et al. 1993, USA [2]	12	30	24 CR and 2 OR	14 (1:13)	36

*CR* closed reduction, *OR* open reduction

### Quality assessment of the included studies

Table 2 summarizes the results of the different domains of study quality adapted from the Newcastle–Ottawa Scale [11]. The retrospective cohort study scored the maximum number of stars on the selection and outcome domains. The study did not specify the extent of the comparability of the hip decompression and no hip decompression groups. The study scored a total of eight out of a maximum of nine stars. The kappa statistics for interobserver agreement on these quality domains was 1.0.

**Table 2:** Quality assessment of the included studies in the meta-analysis (Newcastle–Ottawa Scale)

Domain	Item	Alves et al. 2012 [15]
Selection	Maximum of 4 stars	
	Representativeness of the exposed cohort	*
	Selection of the non-exposed cohort	*
	Ascertainment of exposure	*
	Demonstration that outcome of interest was not present at start of study	*
Comparability	Maximum of 2 stars	
	Comparability of cohorts on the basis of the design or analysis	*
Outcome	Maximum of 3 stars	
	Assessment of outcome	*
	Was follow-up long enough for outcome to occur?	*
	Adequacy of follow-up of cohorts	*

Maximum number of stars is nine for the three domains

### Quantitative results of the meta-analysis

**Fig. 2:**
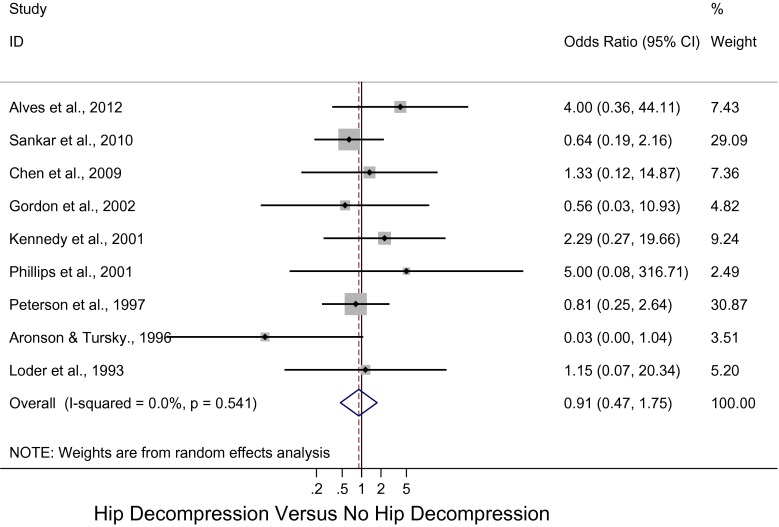
Forest plot: pooled odds ratio (OR) for osteonecrosis in the hip decompression versus no hip decompression groups

Figure 2 displays the cumulative meta-analytic comparison. A random-effects model meta-analysis of the nine studies resulted in an overall pooled OR for osteonecrosis of 0.91 [95 % confidence interval (CI): 0.47, 1.75; *p* = 0.54, *I* ^2^ = 0 %], which suggested a lower rate of osteonecrosis in patients who had hip decompression, but this difference was not statistically significant. The rates of osteonecrosis in the hip decompression and no hip decompression groups were 16.2 and 22.2 %, respectively.

There was no evidence of publication bias from the funnel plots, and the Egger test (*p* = 0.77) was not significant for publication bias.

**Fig. 3:**
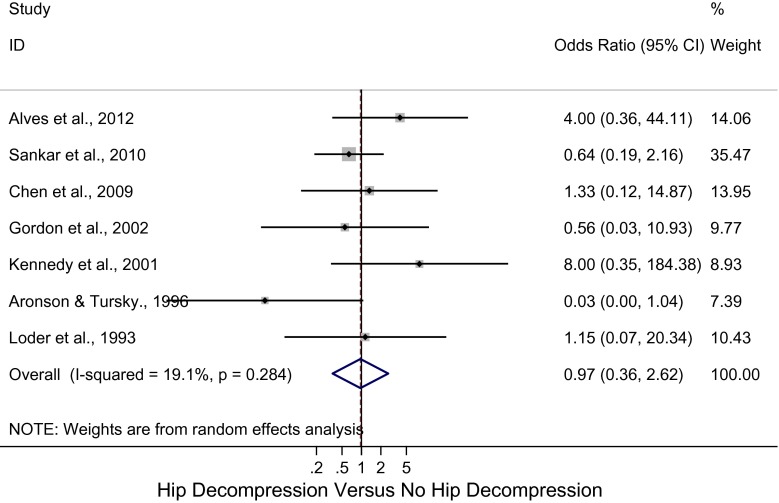
Forest plot: pooled odds ratio (OR) for osteonecrosis in the hip decompression versus no hip decompression groups with no bony procedures

**Fig. 4:**
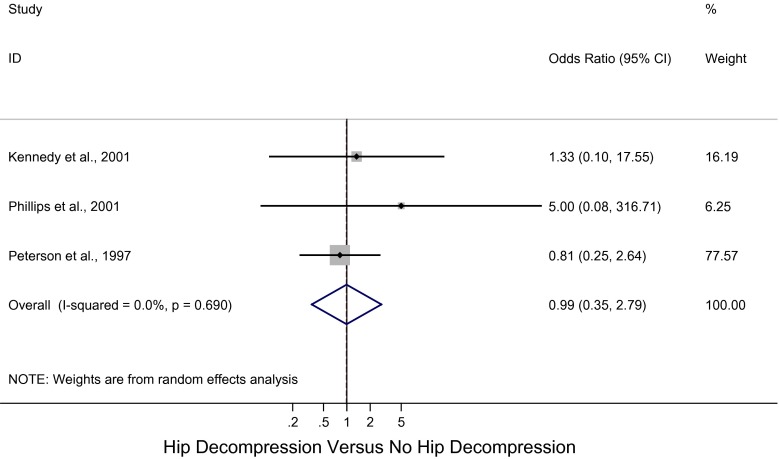
Forest plot: pooled odds ratio (OR) for osteonecrosis in the hip decompression versus no hip decompression groups with bony procedures

Sensitivity analyses revealed a pooled OR for osteonecrosis of 0.97 (95 % CI: 0.36, 2.62; *I* ^2^ = 19.1 %; *p* = 0.28) (Fig. 3) for unstable SCFE that underwent hip decompression with no bony procedures and a pooled OR of 0.99 (95 % CI: 0.35, 2.79; *I* ^2^ = 0 %; *p* = 0.69) (Fig. 4) for unstable SCFE that underwent hip decompression and bony procedures, such as open epiphysiodesis and osteotomies. However, the differences were not significant.

Sensitivity analysis with the inclusion of the study by Kallio et al. [14], which reported a 100 % rate of osteonecrosis in only one patient that underwent hip decompression of a total of 34 patients, revealed only minimal changes in the OR (OR = 1.18; 95 % CI: 0.49, 2.80; *I* ^2^ = 30.9 %; *p* = 0.16) and no significant changes in the overall results.

## Discussion

Hip decompression was associated with a 16.2 % pooled rate of osteonecrosis of the femoral head compared to 22.2 % for no hip decompression in patients with unstable SCFE. The current study revealed no statistically significant difference in the observed rate of osteonecrosis in unstable SCFE when comparing hip decompression and no hip decompression. The results were consistent across different assumptions. The extent to which this statement reflects the true outcome of comparison requires an understanding of the limitations in the current literature and included studies and consideration of the conduct and interpretation of the results of the analyses. The ability to detect a difference is further confounded by the relatively small sample size. Although our study assessed the effect of hip decompression, there was varied reporting regarding the key determinants of unstable SCFE known to influence the rate of osteonecrosis, and none of the included studies reported effect estimates adjusted for these potential confounders.

Intracapsular hip pressure is increased after traumatic femoral neck fractures and, thus, increased intracapsular hip pressure is a known cause of osteonecrosis in femoral neck fractures in children [24, 25]. Decompression of the hip joint can decrease the intracapsular hip pressure and the incidence of osteonecrosis in femoral neck fractures in children [26]. Soto-Hall et al. [27] was the first to measure intracapsular hip pressure in a traumatic SCFE and found a significant increase in the intracapsular hip pressure after reduction (58 mm Hg) compared to the pre-reduction pressure of 12 mm Hg. More recently, Herrera-Soto et al. [10] suggested that unstable SCFE behave like intracapsular hip fractures. Patients with unstable SCFE have intracapsular hip pressures increased to levels higher than those of a compartment syndrome causing a tamponade effect by occluding the venous and arteriole vasculature caused by hematoma formation and effusion. Herrera-Soto et al. [10] observed a 67 % elevation in the intracapsular hip pressure of unstable SCFE after gentle manipulation from 45 to 75 mm Hg. The increase in intracapsular pressure returned to normal values after capsulotomy with a mean pressure of 17 mm Hg. The difference between pre-capsulotomy and post-capsulotomy intracapsular hip pressures was statistically significant. Hence, the authors recommended a capsulotomy to decompress unstable SCFE, especially if gentle manipulation is attempted.

Parsch et al. [28] observed pure blood in 82.8 % of arthrotomies and 17.2 % had a blood-stained rose or clear effusion in a series of 64 consecutive cases of unstable SCFE. In two of the three cases that developed osteonecrosis, blood was drained following hip decompression. Another important variable for the treatment of unstable SCFE is the timing of reduction. Peterson et al. [21] suggested that acute displacement of the femoral epiphysis compromises the blood flow which may be restored by a timely reduction for the unstable SCFE. Both Petersen et al. [21] and Gordon et al. [18] reported lower rates of osteonecrosis in patients treated within 24 h compared to those treated after 24 h. Thus, emergent treatment and hip decompression either with capsulotomy or aspiration is currently recommended in all unstable SCFE to optimize blood flow to the femoral head [4].

There is currently no comparative study demonstrating the superiority of decompression over no decompression in terms of lowering the intracapsular hip pressure of unstable SCFE and preventing osteonecrosis of the femoral head. Surveys of both the European and North American pediatric orthopedic societies showed that hip decompression was recommended by 29 % of European and 35 % of North American pediatric orthopedic surgeons [29, 30]. Our systematic review identified nine studies [2, 15–22] (level III/IV) that compared the rate of osteonecrosis in patients who underwent hip decompression compared to no hip decompression. The one retrospective cohort study included in the analysis was of good methodological quality using the Newcastle–Ottawa Scale with limitations in the comparability domain. Because of the small number and type of included studies, we did not incorporate quality in our sensitivity analysis. The simplest approach is to judge studies on specific domains of quality that are most relevant to the control of bias for that particular study.

Our findings are consistent with the literature, with an overall rate of osteonecrosis of 19.5 %. We found that hip decompression was not associated with a lower rate of osteonecrosis in patients with unstable SCFE. Sensitivity analyses revealed no significant change in the OR of osteonecrosis when no bony and bony procedures were performed with hip decompression in unstable SCFE. However, orthopedic surgeons may consider hip decompression of unstable SCFE as an option that can potentially decompress the intracapsular hip pressure and optimize the blood flow to the femoral head.

Our analysis has a limitation due to the paucity of studies addressing this pivotal issue. There were only nine eligible published studies, but we chose to perform the meta-analysis to provide more generalizable results on the effect estimate. The only outcome measure examined in this meta-analysis was the rate of osteonecrosis. This is a clinically relevant and important outcome and the definition of osteonecrosis was the same amongst the nine studies. Other important factors, such as the time of intervention, the role of reduction, the type of fixation, and post-operative management, could not be controlled for in this analysis and require further study. The limited number of studies addressing these factors permits limited conclusions from the current study. These factors will particularly vary from center to center and variations in clinical skill, implant use, and patient assessment will further confound the results because of these inconsistencies. In addition, others have expressed a concern regarding the different and variable definitions for stable and unstable SCFE [31]. However, the definition of unstable SCFE was uniform amongst the studies included in our analysis. Publication bias was not significant in our meta-analysis.

There has recently been a change of approach to unstable SCFE with urgent reduction, decompression and fixation or open reduction, and fixation using surgical dislocation. The rate of osteonecrosis has been reported to be as low as 8 %, but larger series are required in order to determine the safe approach to unstable SCFE using these surgical techniques [7]. Despite these surgical advancements, Carney et al. [1] concluded that in situ pinning without reduction is the most common treatment worldwide, with the fewest complications.

Our study has only assessed the effect of hip decompression on the rate of osteonecrosis for unstable SCFE, which is one of many factors that may influence osteonecrosis. Hence, the cumulative evidence at present does not indicate an association between hip decompression and a lower rate of osteonecrosis for unstable SCFE. However, orthopedic surgeons may opt to decompress unstable SCFE to decrease intracapsular hip pressure and optimize the blood flow to the femoral head. The results of our meta-analysis are based on observational studies and, thus, further attention should be directed to studies of good methodological quality. Therefore, multicenter prospective cohort studies are required and will be able to answer this question with more certainty and a higher level of evidence.
